# The Concentration of Macrophage‐Derived IL‐1α and IL‐12 in the Peritoneal Fluid of Patients With Endometriosis: A Pilot Case–Control Study

**DOI:** 10.1155/ijin/6669619

**Published:** 2026-10-09

**Authors:** Monika Kopeć, Małgorzata Wojtaś, Aleksandra Kamińska, Michał Kos, Jan Kotarski, Weronika Grochowska, Katarzyna Dos Santos Szewczyk, Marek Gogacz

**Affiliations:** ^1^ Department of Obstetrics and Gynecology and Pregnancy Pathology Independent Public Health Care Department in Łuków Łuków Poland; ^2^ II Chair and Department of Gynecology Medical University of Lublin Lublin 20-954 Poland umlub.pl; ^3^ Independent Laboratory of Cancer Diagnostics and Immunology Medical University in Lublin Lublin 20-093 Poland umlub.pl; ^4^ Department of Pharmaceutical Botany Medical University of Lublin Lublin 20-093 Poland umlub.pl

**Keywords:** cytokines, endometriosis, inflammation, macrophages, peritoneal fluid

## Abstract

It is supposed that the primary or secondary dysfunction of macrophages may play an essential role in the pathogenesis of endometriosis. It is postulated that macrophage‐derived cytokines and signaling molecules, such as IL‐1 (interleukin), IL‐6, and IL‐12, tumor necrosis factor‐alpha (TNF‐α), interferon‐gamma (IFN‐γ), and MCP‐1 (monocyte chemoattractant protein) may be involved in the process of endometriosis formation. This pilot study included 47 patients who underwent diagnostic–therapeutic laparoscopy at the II Department of Gynecology, Medical University of Lublin, Poland. Peritoneal fluid was obtained from patients in both the reference and study groups. The number and viability of the cells were assessed. The cells were labeled with monoclonal antibodies and assessed using flow cytometry. Interleukins of macrophage origin in the peritoneal fluid were determined using the enzyme‐linked immunosorbent assay (ELISA) test. The peritoneal fluid showed fundamental changes in the concentration of the studied cytokines. We recorded the peritoneal fluid volume for each patient (median ∼5 mL in the endometriosis group vs. ∼4 mL in the reference group) and noted that a larger volume could dilute cytokine levels; cytokine concentrations were thus interpreted per mL of fluid. More than twice as high median IL‐12 levels were detected in women in the reference group than in the study group. High levels of IL‐1α and low levels of IL‐12 in the study group compared to the reference group suggest an immunological component in the etiopathogenesis of endometriosis, although this pilot, exploratory study (*n* = 47) cannot establish this relationship definitively. All endometriosis cases were laparoscopically confirmed, but histopathological confirmation was obtained in only 20 of 28 cases (71.4%); the remaining 8 patients had superficial Stage I lesions that were not biopsied. This study aimed to assess the concentrations of IL‐1α and IL‐12 in the PF of women with endometriosis compared to those with idiopathic infertility.

## 1. Introduction

Endometriosis remains a debilitating and underdiagnosed condition, which, owing to numerous nonspecific symptoms (fatigue, pelvic pain, bloating, dyspareunia, dysuria, and constipation), significantly delays the final diagnosis by an average of 7–12 years. In total, 20%–25% of patients may be asymptomatic [1–4]. Pain is one of the most frequently reported symptoms [5]. Over time, and with increasing disease severity, affected women may manifest stress, somatization, and depression [6, 7]. Despite decades of research and its frequent occurrence, the etiopathogenesis of endometriosis remains unexplained [8, 9]. Early menarche, short menstrual cycles (< 27 days), long menstruation (> 7 days), and a family history of endometriosis are considered risk factors. The involvement of genetic, hormonal, environmental, and immunological factors has been identified, with the immune system playing an important role [10–12]. In 1981, Dmowski first proposed that immune system disorders appear at almost every stage of disease development [13]. Macrophages are key components responsible for recognizing and disposing of foreign and damaged cells in the peritoneal cavity [14]. In patients with endometriosis, an increased number of activated peritoneal macrophages with reduced phagocytic capacity have been observed. A proteomics‐based theory describes a peritoneal protein (comparable to haptoglobin) that adheres to macrophages, decreases their phagocytic effect, and stimulates IL‐6 production [14]. In line with this, several inflammatory mediators are elevated in the peritoneal fluid (PF) of women with endometriosis, including monocyte chemoattractant protein‐1 (MCP‐1), IL‐8, and IL‐1β. Neovascularization is essential for ectopic lesion development; activated peritoneal macrophages secrete vascular endothelial growth factor (VEGF), enhancing angiogenesis in inflamed areas [15–17]. The peritoneal microenvironment is rich in prostaglandins, which contribute to pain sensation. Elevated estrogen levels increase cyclooxygenase‐2 (COX‐2) activity, stimulating prostaglandin E2 (PGE_2_) secretion, which directly stimulates nerve endings and pain signaling. The principal macrophage‐derived mediators and their cellular targets are summarized in Figure 1.

**FIGURE 1:**
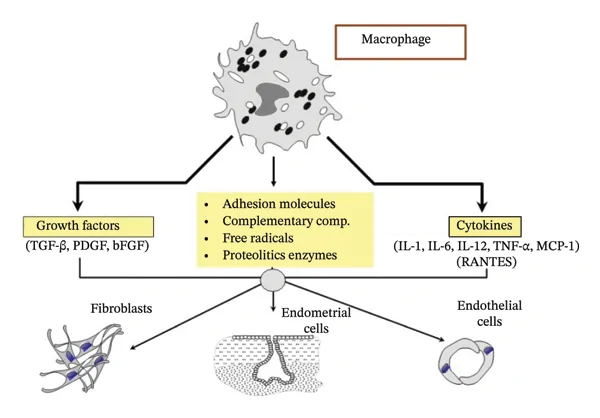
Activated peritoneal macrophages and their secreted components [18].

Endometriosis is difficult to prevent because of the lack of known causes. The main aim of this study was to review the immune system’s role, with a special focus on macrophage function in endometriosis, and to evaluate the concentration of IL‐1α and IL‐12 in the PF of women with endometriosis in relation to a reference group (women with idiopathic infertility) [5, 19–21]. We concentrated on IL‐1α and IL‐12 because these two macrophage‐derived interleukins represent complementary aspects of endometriosis immunology: IL‐1α is a key proinflammatory cytokine implicated in endometriosis‐associated inflammation, whereas IL‐12 is crucial for cell‐mediated immune responses (driving T helper 1 and natural killer cell activity), which are often impaired in endometriosis [22–25]. In contrast, other macrophage cytokines, such as IL‐6 and tumor necrosis factor‐alpha (TNF‐α), have been more extensively studied and are consistently found to be elevated in endometriosis [26], while interferon‐gamma (IFN‐γ) levels in PF are notably suppressed in affected women [25, 27]. MCP‐1 levels are also increased, correlating with macrophage recruitment to the peritoneal cavity [28]. By focusing on IL‐1α and IL‐12, we aimed to explore the balance between excessive inflammatory stimulation and deficient cytotoxic immune surveillance in the peritoneal microenvironment of endometriosis.

In healthy menstruating women, straw‐colored, aqueous‐serous PF is produced at approximately 20 mL per day, and the volume fluctuates with the menstrual cycle phase [29]. PF is produced primarily by the peritoneum and ovaries, and its composition includes follicular fluid, retrograde menstrual fluid, immune cells, and cellular debris [30]. Immunocompetent cells account for more than 80% of PF cells, with macrophages comprising over 50% of them [31]. In the peritoneal cavity, monocytes and macrophages are secretory and phagocytic, being the main source of many cytokines (including IL‐1α and IL‐12) that play fundamental roles in cellular and humoral immune responses [22]. Activated macrophages express HLA‐DR (an MHC class II antigen) on their surface, which is a marker of activation that can be measured by flow cytometry [32]. These peritoneal macrophages are believed to be the first line of defense against endometriosis [33, 34]. Activated peritoneal macrophages are the main source of IL‐1α and IL‐12 in the PF [35, 36]. Biologically active IL‐1 (also known as lymphocyte‐activating factor) is a ∼17 kDa polypeptide produced primarily by activated monocytes/macrophages [35, 36]. IL‐1 exerts diverse effects, including the activation of T lymphocytes (inducing IL‐2 receptor expression and IL‐2 gene), activation of fibroblasts, promotion of angiogenesis, and induction of apoptosis in immune cells [37, 38] (Figure 2).

**FIGURE 2:**
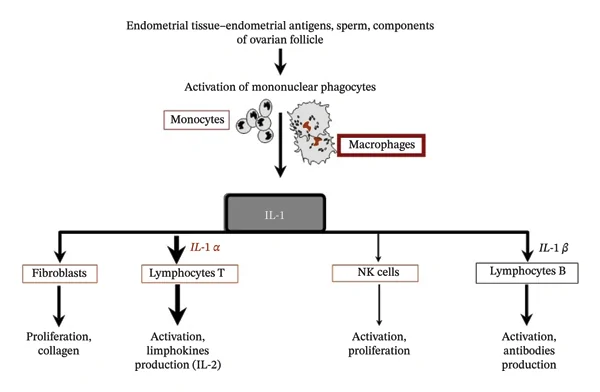
Macrophages interact with other immune cells [39].

IL‐12, also known as natural killer cell stimulatory factor (NKSF) or cytotoxic lymphocyte maturation factor (CLMF), is a heterodimeric cytokine produced mainly by monocytes/macrophages [27]. The biological action of IL‐12 is mainly associated with stimulating the proliferative activity and cytotoxicity of NK cells and cytotoxic T lymphocytes and inducing IFN‐γ production [27]. IL‐12 enhances antigen presentation, phagocytosis, and nitric oxide production. It can have more potent effects on NK cell cytotoxicity than IFN‐γ or IL‐2, and it induces interferon‐inducible protein‐10 (IP‐10), which strongly inhibits angiogenesis [27]. Given these roles, a deficiency of IL‐12 in the peritoneal cavity could hinder cytotoxic immune responses (e.g., reduced IFN‐γ production and NK cell activity) and allow endometriotic lesions to persist [23–25]. Conversely, excess IL‐1α can intensify local inflammation and angiogenesis, promoting lesion establishment (Figure 3).

**FIGURE 3:**
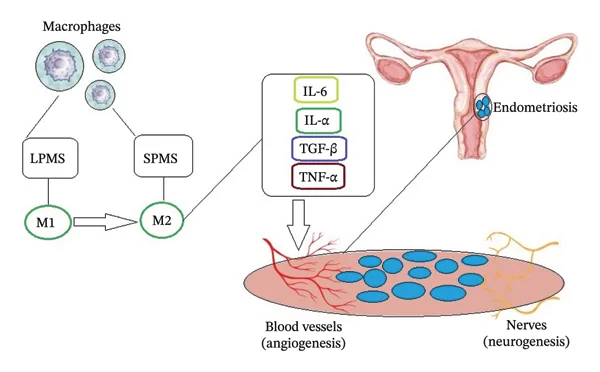
Inflammatory microenvironment in endometriotic lesions. Large peritoneal macrophages (LPMs), specialized proresolving mediators, IL‐6 (Interleukin 6), IL‐1α (Interleukin 1α), tumor growth factor‐β (TGF‐β), and tumor necrosis factor‐α [40].

## 2. Material and Methods

### 2.1. Patients

A total of 47 patients undergoing diagnostic‐therapeutic laparoscopy at the II Department of Gynecology, Medical University of Lublin, Poland, were included in the study. Twenty‐eight patients were assigned to the endometriosis group based on laparoscopic findings according to the revised American Society for Reproductive Medicine (rASRM) classification. The stage distribution was as follows: Stage I (minimal), *n* = 8; Stage II (mild), *n* = 7; Stage III (moderate), *n* = 9; and Stage IV (severe), *n* = 4.

All 28 endometriosis cases were laparoscopically confirmed. Histopathological confirmation was obtained in 20 of the 28 cases (71.42%). The remaining 8 patients had superficial Stage I peritoneal lesions with a characteristic laparoscopic appearance and did not undergo biopsy because of their small size. Consequently, although all endometriosis cases were laparoscopically confirmed, not all lesions underwent histopathological verification. Histopathology remains the diagnostic gold standard, and incomplete histological confirmation should be considered a limitation of the study.

The reference group comprised women with idiopathic infertility who demonstrated no visible endometriotic lesions during laparoscopic evaluation (*n* = 19). These patients had no anatomical or endocrine causes of infertility and no macroscopic evidence of endometriosis during surgery. Patients with ovarian endometriomas, dense pelvic adhesions, deep infiltrating lesions, or other laparoscopic findings highly suggestive of endometriosis were not included in the reference group. Occasional minor filmy adhesions observed in some control patients were considered nonspecific inflammatory or incidental findings because they were not associated with characteristic endometriotic lesions or other diagnostic features of endometriosis. However, random peritoneal biopsies were not performed in the reference group. Therefore, the presence of occult microscopic endometriosis cannot be completely excluded. Consequently, the reference group should be interpreted as laparoscopically negative rather than histologically confirmed disease‐free controls. Laparoscopy was performed during the early to midfollicular phase of the menstrual cycle (typically between cycle Days 5 and 12) to minimize hormonal variability in immune parameters. The age of the patients ranged from 22 to 45 years. All participants had regular menstrual cycles lasting 25–34 days. None of the patients had used hormonal therapy for at least 3 months before surgery, and none used intrauterine contraceptive devices. Patients with chronic inflammatory, autoimmune, or immunomodulating disorders, as well as patients receiving immunosuppressive treatment, were excluded from the study. All participants provided written informed consent, and the study protocol was approved by the Institutional Ethics Board (KE‐0254/290/2003).

### 2.2. Biological Material

PF was obtained from all patients immediately after entering the abdominal cavity and before any surgical manipulation in order to minimize procedure‐related contamination. Under direct laparoscopic visualization, PF was aspirated from the pouch of Douglas and other dependent peritoneal areas using sterile suction cannulas. The total volume of PF collected from each patient was measured and recorded. Samples showing visible fresh blood contamination caused by trocar insertion or surgical trauma were predefined for exclusion because blood‐derived cytokines may significantly influence cytokine measurements in PF. In the present study, none of the collected samples demonstrated visible blood contamination. Therefore, no patients were excluded for this reason. The collected PF was divided into two portions: one portion was transferred into heparinized tubes for cellular analysis, while a 2 mL aliquot was collected into EDTA‐treated tubes for cytokine determination. Samples were centrifuged for 10 min at 700 × *g*, and the cell‐free supernatants were aliquoted and stored at −80°C until further analysis. The volume of PF obtained from each patient was measured and recorded. Cytokine concentrations were expressed as pg/mL of PF and were not additionally normalized to total aspirated fluid volume.

### 2.3. Isolation of PF Mononuclear Cells

Mononuclear cells (primarily leukocytes) from PF were isolated by density gradient centrifugation using Lymphoprep (Axis‐Shield, Norway) within 1 h of collection. The PF was diluted 1:1 with sterile 0.9% buffered saline solution (Biomed) and carefully layered over the Lymphoprep solution in 15 mL conical tubes. After centrifugation, the mononuclear cell layer was harvested, washed, and analyzed using flow cytometry (Figure 4).

**FIGURE 4:**
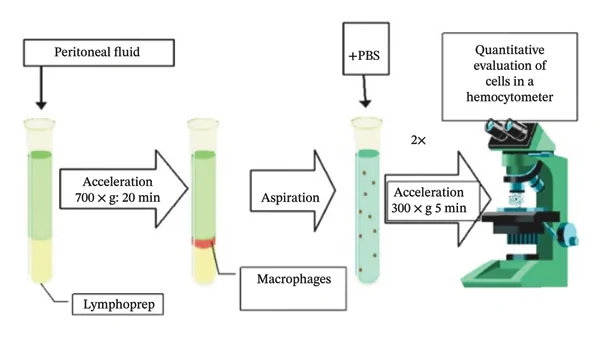
Isolation of macrophages from PF.

### 2.4. Flow Cytometry

The number and viability of PF cells were assessed using trypan blue exclusion. For immunophenotyping, cells were labeled with fluorochrome‐conjugated monoclonal antibodies against CD14 and HLA‐DR (anti–CD14‐FITC [Cat. No. MHCD1401, CALTAG, UK] or anti–CD14‐TC [Cat. No. MHCD1406, CALTAG, UK]; anti–HLA‐DR‐APC/Cy7 [Clone L203, Cat. No. FAB4869APCCY7, R&D Systems, USA]). Matched fluorochrome‐conjugated isotype control antibodies were used to define positivity thresholds (mouse IgG1/IgG1‐FITC/PE [Cat. No. M1F2aP, CALTAG, UK] and antihuman IgG‐FITC [Cat. No. 555786, BD Pharmingen, USA]). HLA‐DR/CD14 immunophenotyping was performed on freshly isolated PF mononuclear cells immediately after their isolation. No short‐term ex vivo culture or stimulation was performed prior to flow cytometric analysis. After labeling, cells were incubated for 30 min at 4°C in the dark, washed, and fixed in 1% paraformaldehyde. Data were acquired on a FACSCalibur flow cytometer (Becton Dickinson, USA) equipped with standard compensation controls (single‐stained cells for each fluorochrome [CD14‐FITC and HLA‐DR‐APC/Cy7 alone]). Macrophages were identified using a sequential gating strategy: an initial forward scatter (FSC‐A) versus side scatter (SSC‐A) gate was used to exclude debris, followed by a doublet‐exclusion gate (FSC‐H vs. FSC‐A) and, where applicable, a viability gate to exclude dead cells (propidium iodide). Macrophages were then defined within the mononuclear cell gate as CD14^+^ events, and HLA‐DR expression was assessed within this CD14^+^ population using the isotype/FMO‐defined positivity threshold described above. Throughout our manuscript, the parameter “percentage of macrophages” refers specifically to the proportion of CD14^+^ events among gated peritoneal mononuclear cells (i.e., the CD14^+^ fraction of the FSC/SSC‐gated, doublet‐ and dead‐cell–excluded mononuclear population); it does not denote CD14^+^/HLA‐DR^+^ double‐positive cells, nor HLA‐DR^+^ cells considered alone. HLA‐DR expression, assessed within the CD14^+^ (macrophage) gate as described above, was used in this study primarily to support macrophage identification and gating quality; a separate, quantitative percentage of HLA‐DR^+^ cells within the CD14^+^ population was not systematically tabulated for each patient and is therefore not reported as a distinct result (Figure 5).

**FIGURE 5:**
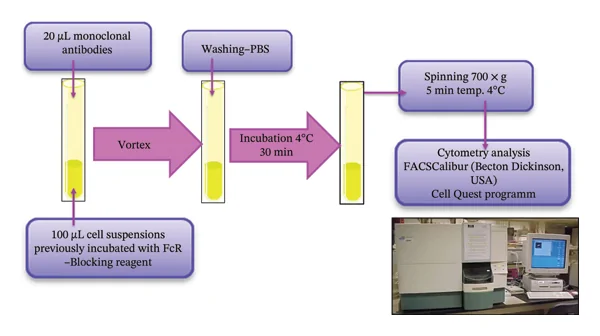
Assessment of macrophage cell marker expression.

### 2.5. Cytokine Determination (Enzyme‐Linked Immunosorbent Assay [ELISA])

Macrophage‐derived cytokines in PF, including IL‐1α and IL‐12, were measured using commercially available ELISA kits according to the manufacturers’ protocols. IL‐1α concentrations were determined using the Human IL‐1α Quantikine ELISA kit (R&D Systems, USA), with a reported analytical sensitivity below 1 pg/mL according to the manufacturer’s specifications. IL‐12 p70 concentrations in PF were determined using the Human IL‐12 p70 Quantikine HS (high sensitivity) ELISA kit (Cat. No. HS120, R&D Systems, Minneapolis, MN, USA). The assay was used because of its high analytical sensitivity, appropriate for the low IL‐12 concentrations detected in PF. Briefly, 100 μL of thawed PF supernatant was added in duplicate to microplate wells precoated with capture antibodies and subsequently incubated with detection antibodies and substrate solution. Absorbance was measured at the recommended wavelength using an ELx800 microplate reader (BioTek Instruments, USA). Standard curves were generated using the cytokine standards supplied with the kits, and cytokine concentrations were calculated accordingly. The assays demonstrated good analytical reliability, with intra‐assay coefficients of variation (CVs) of approximately 1%–4% and inter‐assay CVs of approximately 4%–8% within the measured concentration ranges. These values were based on manufacturer validation data and confirmed by duplicate laboratory measurements performed during the study. The lower limit of detection (LLOD) and lower limit of quantification (LOQ) reported by the manufacturer were (1 pg/mL and 3.9 pg/mL, respectively, per the manufacturer’s datasheet for the Quantikine Human IL‐1α/IL‐1F1 ELISA kit [Cat. No. DLA50, R&D Systems]) for IL‐1α and (0.5 pg/mL and 0.8 pg/mL, respectively, consistent with the Quantikine HS Human IL‐12 p70 ELISA kit [Cat. No. HS120, R&D systems]) for IL‐12. Six of the 47 IL‐12 measurements (all in the endometriosis group; Table 1) yielded concentrations at or below the manufacturer‐defined LOQ of 0.8 pg/mL, comprising five values of 0.2 pg/mL at the lower asymptote of the standard curve and one value of 0.5 pg/mL at the nominal LLOD. Rather than substituting these with an arbitrary value (e.g., 0 pg/mL), the concentrations were back‐calculated directly from the four‐parameter logistic standard curve, as is standard practice for high‐sensitivity ELISA assays, and retained as continuous data. Values in this sub‐LOQ range carry greater analytical imprecision than values above the LOQ and should be interpreted with corresponding caution. To confirm that inclusion of these six sub‐LOQ values did not drive the main between‐group result, we performed a sensitivity analysis in which the endometriosis‐versus‐reference comparison for IL‐12 was repeated after (i) excluding the six sub‐LOQ values (*n* = 22 vs. 19) and (ii) substituting them with LOQ/2 (0.4 pg/mL) or LLOD/2 (0.25 pg/mL). IL‐12 concentrations remained significantly lower in the endometriosis group under every approach (Mann–Whitney *U* = 61.5, *p* < 0.001 for both imputation strategies; *p* < 0.001 after exclusion of sub‐LOQ values), confirming that handling of these low values did not affect the main result.

**TABLE 1:** Percentage of macrophages in peritoneal fluid obtained from the study and reference groups, and concentration of IL‐1α and IL‐12 in peritoneal fluid obtained from the study and reference groups (pg/mL).

L.p.	Group (1—patients with endometriosis; 2—reference group patients)	IL‐1α (pg/mL)	IL‐12 (pg/mL)	Percentage of macrophages in peritoneal fluid (%)
1	1	8.2	0.2	86.0
2	1	7.5	0.2	84.6
3	1	7.5	4.5	85.0
4	1	5.4	5.6	83.5
5	1	5.7	0.5	81.7
6	1	37.1	2.7	72.4
7	1	7.5	2.9	88.6
8	1	9.9	3.4	90.3
9	1	10.8	2.7	82.3
10	1	7.2	0.9	76.4
11	1	35.3	2.0	89.8
12	1	8.7	2.2	74.4
13	1	6.6	1.9	81.2
14	1	6.3	3.8	87.2
15	1	13.8	1.6	88.1
16	1	6.6	2.2	76.8
17	1	9.0	1.9	77.7
18	1	6.3	0.2	87.2
19	1	7.5	2.0	92.2
20	1	7.5	0.2	90.4
21	1	6.0	0.2	86.7
22	1	6.6	1.1	83.5
23	1	5.7	2.7	78.7
24	1	7.5	1.6	83.5
25	1	4.8	3.6	68.9
26	1	6.3	5.4	64.6
27	1	6.9	2.9	65.8
28	1	8.7	1.1	75.4
29	2	4.8	3.8	47.8
30	2	4.5	6.5	40.5
31	2	9.0	4.2	68.6
32	2	4.5	3.8	57.1
33	2	6.9	4.2	52.5
34	2	5.9	3.8	41.0
35	2	4.4	4.2	73.5
36	2	4.5	4.2	46.9
37	2	6.1	3.8	57.6
38	2	6.1	3.1	67.5
39	2	5.4	2.7	52.8
40	2	5.9	3.6	49.7
41	2	6.1	5.4	54.6
42	2	4.8	3.8	45.2
43	2	6.9	3.6	45.1
44	2	5.4	5.6	62.8
45	2	5.4	5.8	54.1
46	2	5.8	4.6	64.2
47	2	6.2	3.8	57.3

### 2.6. Statistical Analysis

Statistical analyses were performed using Statistica 13.1 (TIBCO Software Inc., Palo Alto, CA, USA). Continuous variables were assessed for normality using the Shapiro–Wilk test. Because IL‐1α, IL‐12, and macrophage percentage demonstrated non‐normal distributions, nonparametric analyses were performed. Comparisons between the endometriosis and reference groups were conducted using the Mann–Whitney *U* test with continuity correction. Results are presented as medians with interquartile ranges (IQRs), ranges, *p*‐values, and 95% confidence intervals where appropriate. Statistical significance was set at *p* < 0.05. Associations between variables were assessed using Spearman’s rank correlation coefficient (*ρ*). Pearson correlation analysis was not applied because the assumptions of normality, linearity, and homoscedasticity were not satisfied. Correlations were analyzed between IL‐1α and IL‐12 concentrations, IL‐1α concentration and macrophage percentage, and IL‐12 concentration and macrophage percentage in PF. To supplement the *p*‐values from the Mann–Whitney *U* test, between‐group differences for macrophage percentage, IL‐1α, and IL‐12 were additionally characterized using the Hodges–Lehmann estimator of the median difference with its associated 95% confidence interval, and effect size was quantified using the rank‐biserial correlation (equivalent to Cliff’s delta). To explore the potential value of macrophage percentage, IL‐1α, and IL‐12 as discriminatory markers between the endometriosis and reference groups, receiver operating characteristic (ROC) curve analysis was performed for each variable, with the area under the curve (AUC) reported together with a bootstrap‐derived (2000 resamples) 95% confidence interval as an internal estimate of precision, and the Youden index used to identify an optimal cutoff with corresponding sensitivity and specificity. These ROC/AUC analyses are presented as exploratory, hypothesis‐generating estimates of discriminatory performance in this pilot cohort; they represent apparent (in‐sample) rather than externally validated performance, and independent validation in an external cohort is required before any diagnostic cutoff can be considered for clinical use.

## 3. Results

### 3.1. Patient Characteristics

The baseline demographic and clinical characteristics of the study population are summarized in Table 2. The endometriosis and reference groups were comparable with respect to age, menstrual cycle phase at surgery, and reproductive history. All laparoscopic procedures were performed during the early to midfollicular phase of the menstrual cycle (cycle Days 5–12) according to the study protocol. The distribution of endometriosis stages according to the revised rASRM classification is also presented. The mean age of women with endometriosis was 30.5 ± 5.4 years and did not differ significantly from that of the reference group (29.8 ± 4.9 years, *p* = 0.72). The proportion of nulliparous women was similar in both groups (89% vs. 89%, *p* = 0.99). The volume of PF obtained during laparoscopy ranged from approximately 2 mL to 15 mL. Patients with endometriosis demonstrated a slightly higher median PF volume than controls (5 mL vs. 4 mL, respectively), although the difference was not statistically significant (*p* = 0.18). Because cytokine concentrations were reported per mL of fluid, differences in total fluid volume may still represent a potential dilution‐related confounding factor.

**TABLE 2:** Baseline demographic and clinical characteristics of patients with endometriosis and the reference group.

Characteristic	Endometriosis (*n* = 28)	Control (*n* = 19)	*p*‐value
Age (years)	30.5 ± 5.4	29.8 ± 4.9	0.72 (ns)
Cycle day of surgery (median [range])	8 [5–12]	8 [5–12]	N/A^∗^
Nulliparous, *n* (%)	25 (89%)	17 (89%)	0.99 (ns)
Peritoneal fluid volume (median [range], mL)	5 [2–15]	4 [2–12]	0.18
Stage I, *n* (%)	8 (28.6%)	N/A	—
Stage II, *n* (%)	7 (25.0%)	N/A	—
Stage III, *n* (%)	9 (32.1%)	N/A	—
Stage IV, *n* (%)	4 (14.3%)	N/A	—

*Note:* Values are expressed as mean ± SD or median (range). N/A, not applicable (the characteristic was identical to that of the study design), ns = not statistically significant.

^∗^All surgeries were performed in the early‐mid follicular phase (Days 5–12 of the menstrual cycle).

Laparoscopic findings confirmed pelvic endometriosis in 28 patients, distributed according to rASRM stage as follows: Stage I (minimal), *n* = 8; Stage II (mild), *n* = 7; Stage III (moderate), *n* = 9; and Stage IV (severe), *n* = 4. No cases of deeply infiltrating endometriosis (e.g., bowel or rectovaginal lesions with > 5 mm penetration) were observed.

Comparisons of immunological parameters between patients with endometriosis (Group 1) and the reference group (Group 2) were performed using the Mann–Whitney *U* test. Statistically significant differences were observed for all the analyzed variables.

### 3.2. PF Cell Profile

The percentage of macrophages in PF was significantly higher in women with endometriosis than in controls [median 83.5%, IQR 76.7–87.2 vs. 54.1%, IQR 47.4–60.2; Mann–Whitney *U* = 8.0, *p* < 0.001]. The Hodges–Lehmann estimate of the median between‐group difference was 27.7% points (95% CI: 21.8–33.1), with a rank‐biserial correlation (Cliff’s delta) of −0.97, indicating a very large effect size. Here, “percentage of macrophages” refers to the proportion of CD14^+^ events among gated peritoneal mononuclear cells, not CD14^+^/HLA‐DR^+^ double‐positive cells or HLA‐DR^+^ cells alone. This finding indicates a significantly higher proportion of macrophages among peritoneal mononuclear cells in women with endometriosis, consistent with increased macrophage recruitment and/or accumulation in the peritoneal microenvironment. Because HLA‐DR positivity within the CD14^+^ gate was not separately reported as a quantitative activation index in this dataset, this finding should not, on its own, be interpreted as direct evidence of increased macrophage activation.

### 3.3. Cytokine Concentrations in PF

The median IL‐1α concentration was significantly higher in women with endometriosis than in controls [7.5 pg/mL, IQR 6.3–8.7, range 4.8–37.1, 95% CI 6.6–7.9 vs. 5.8 pg/mL, IQR 4.8–6.1, range 4.4–9.0, 95% CI 4.8–6.1; Mann–Whitney *U* = 83.0, *p* < 0.001]. The Hodges–Lehmann estimate of the median between‐group difference was 1.7 pg/mL (95% CI: 0.9–2.7), with a rank‐biserial correlation (Cliff’s delta) of −0.69, indicating a large effect size.

In contrast, the median IL‐12 concentration was significantly lower in the endometriosis group than in the control group (2.0 pg/mL, IQR 1.05–2.9, range 0.2–5.6, 95% CI 1.4–2.7 vs. 3.8 pg/mL, IQR 3.8–4.4, range 2.7–6.5, 95% CI 3.8–4.2; Mann–Whitney *U* = 61.5, *p* < 0.001). The Hodges–Lehmann estimate of the median between‐group difference was −2.2 pg/mL (95% CI: −2.9 to −1.4), with a rank‐biserial correlation (Cliff’s delta) of 0.77, indicating a large effect size. These findings indicate a distinct inflammatory profile in women with endometriosis, characterized by elevated IL‐1α levels and reduced IL‐12 concentrations in PF.

*Correlation analyses*: Associations among IL‐1α, IL‐12, and the percentage of macrophages were examined separately in both groups using Spearman’s rank correlations. In the endometriosis group, no statistically significant associations were observed between IL‐1α concentration and macrophage percentage (*ρ* = 0.205, *p* > 0.05) (Figure 6), IL‐12 and macrophages (*ρ* = −0.246, *p* > 0.05) (Figure 7), or IL‐1α and IL‐12 concentrations (*ρ* = −0.106, *p* > 0.05) (Figure 8). Similarly, in the reference group, no statistically significant correlations were found between IL‐1α concentration and macrophage percentage (*ρ* = 0.166, *p* > 0.05) (Figure 9), IL‐12 concentration and macrophage percentage (*ρ* = 0.099, *p* > 0.05) (Figure 10), or IL‐1α and IL‐12 concentrations (*ρ* = −0.233, *p* > 0.05) (Figure 11).

**FIGURE 6:**
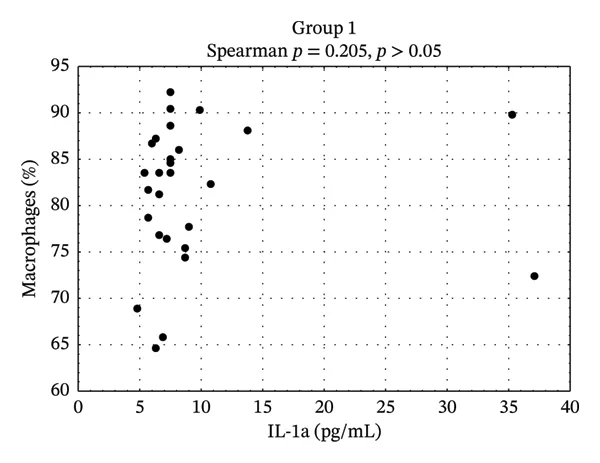
Correlation between IL‐1α and the percentage of macrophages in the peritoneal fluid of patients with endometriosis (Group 1).

**FIGURE 7:**
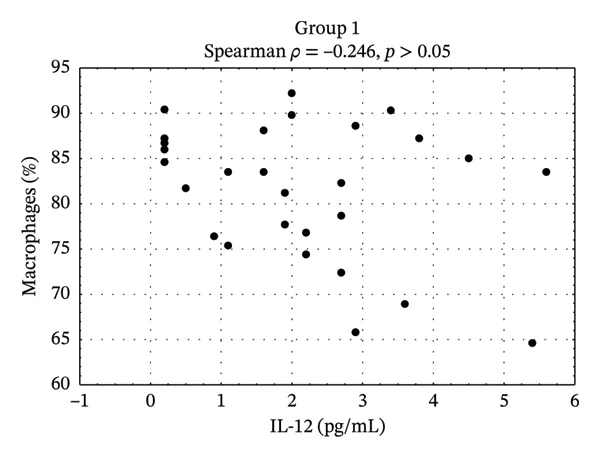
Correlation between IL‐12 concentration and percentage of macrophages in the peritoneal fluid of patients with endometriosis (Group 1).

**FIGURE 8:**
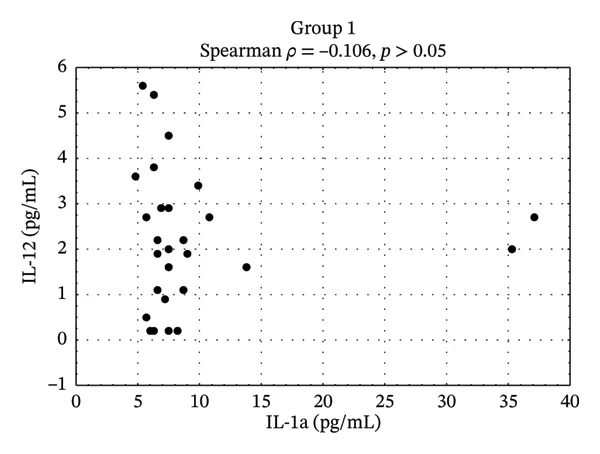
Correlation between IL‐1α and IL‐12 levels in the peritoneal fluid of patients with endometriosis (Group 1).

**FIGURE 9:**
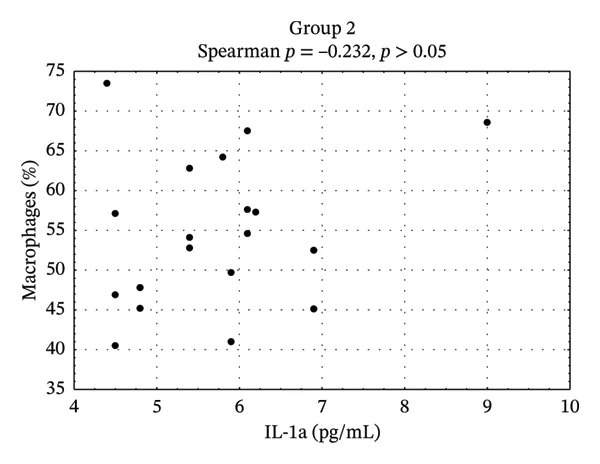
Correlation between IL‐1α and the percentage of macrophages in the peritoneal fluid of the reference group (Group 2).

**FIGURE 10:**
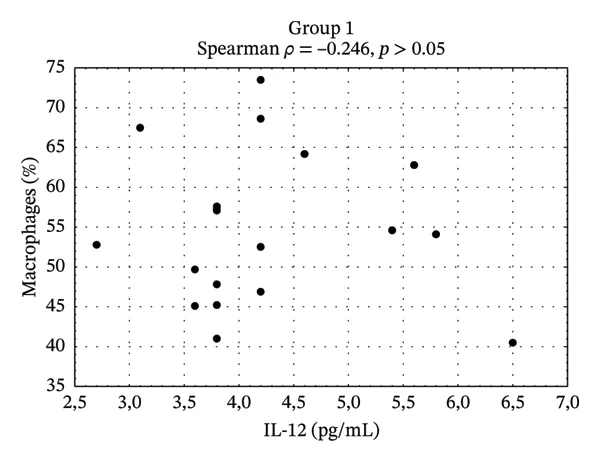
Correlation between IL‐12 concentration and percentage of macrophages in the peritoneal fluid of the reference group (Group 2).

**FIGURE 11:**
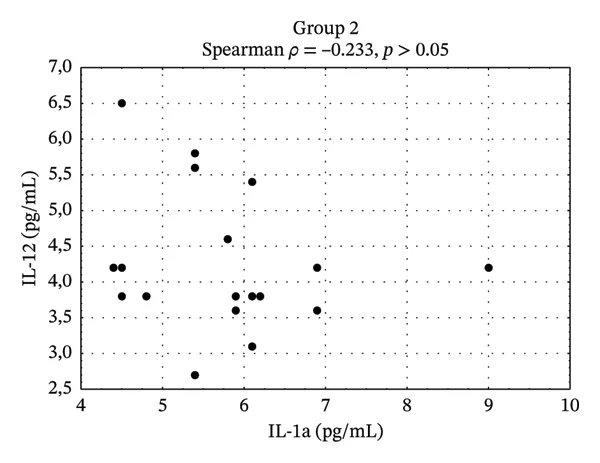
Correlation between IL‐1α and IL‐12 levels in the peritoneal fluid of the reference group (Group 2).

Correlation analyses did not demonstrate statistically significant associations between cytokine concentrations and macrophage percentage in either group.

*Exploratory discriminatory performance*: Because elevated IL‐1α and reduced IL‐12 have been proposed as candidate biomarkers of endometriosis in this cohort, ROC analysis was performed for macrophage percentage, IL‐1α, and IL‐12 as exploratory classifiers distinguishing women with endometriosis from the reference group. Macrophage percentage showed the strongest apparent discriminatory performance (AUC = 0.985, bootstrap 95% CI: 0.953–1.000; Youden‐optimal cutoff = 68.9%, sensitivity = 93%, specificity = 95%), followed by IL‐12 (AUC = 0.884, 95% CI: 0.778–0.976; cutoff = 2.9 pg/mL, sensitivity = 79%, specificity = 95%) and IL‐1α (AUC = 0.844, 95% CI: 0.715–0.951; cutoff = 6.3 pg/mL, sensitivity = 82%, specificity = 84%). These AUC and cutoff estimates reflect apparent (in‐sample, bootstrap‐supported) performance in this pilot cohort only. No external or independent validation cohort was available, and these values should therefore be regarded as hypothesis‐generating rather than as validated diagnostic thresholds. Prospective validation in an independent cohort, ideally with prespecified cutoffs, is required before any of these markers could be considered for clinical diagnostic use.

Table 1 summarizes the above findings, listing the percentage of macrophages and the concentrations of IL‐1α and IL‐12 for each patient in both groups.

## 4. Discussion

Many studies suggest that peritoneal immune system disorders play a key role in the development of endometriosis [41, 42]. In the peritoneum of women with endometriosis, macrophages, which are key cells of the innate immune system, are highly activated and present in increased numbers, accompanied by elevated levels of proinflammatory mediators that alter the microenvironment and create conditions favoring the development of endometriosis [43]. In the present study, we documented a ∼1.5‐fold higher total concentration (proportion) of macrophages in the PF of patients with endometriosis than in the reference group, consistent with this concept. This observation agrees with the data published by Akoumen et al. [28], who similarly found an increased peritoneal macrophage count in endometriosis along with elevated MCP‐1 levels (a chemokine that recruits macrophages). Thao et al. [44] also reported that the expression of HLA‐DR (an activation marker) was significantly higher in PF macrophages from women with confirmed endometriosis than in controls. In our study, HLA‐DR expression was assessed by flow cytometry within the CD14^+^ gate as part of the macrophage identification strategy. The “percentage of macrophages” reported in the Results and Table 1 corresponds to the CD14^+^ fraction of gated mononuclear cells and does not represent the proportion of HLA‐DR^+^ cells. Because a separate, patient‐level quantification of the HLA‐DR^+^ percentage within the CD14^+^ gate was not systematically reported for this cohort, our data cannot be directly compared with those of Thao et al. [44], who quantified HLA‐DR expression as a discrete activation readout. HLA‐DR on macrophages is generally understood as a marker of antigen‐presenting capacity and, more broadly, of an activated phenotype: its expression is important for the induction of a humoral immune response (antigen presentation to helper T cells), in addition to its role in cellular immunity. In the absence of a quantitative HLA‐DR^+^ activation measure in the present dataset, however, our finding of an increased CD14^+^ macrophage proportion in endometriosis is best interpreted as evidence of macrophage accumulation. The further inference of increased macrophage activation remains a biological hypothesis that should be confirmed with a dedicated activation readout in future studies.

Peritoneal macrophages participate in endometrial debris clearance, tissue repair, and regeneration during the menstrual cycle. During inflammatory responses, they play a dual role: initially secreting inflammatory mediators at an early stage (characterized by high expression of proinflammatory factors, including TNF‐α, IL‐1α, and NO) and later producing immunosuppressive factors, such as IL‐10, IL‐4, IL‐13, and TGF‐β [45]. To balance the tissue‐damaging potential of inflammatory macrophages, they either undergo apoptosis or switch to an anti‐inflammatory (M2) phenotype, thereby reducing the proinflammatory response. The balance between macrophage activation and timely apoptosis or polarization is important for maintaining peritoneal homeostasis [46]. Disruption of this balance can lead to persistent inflammatory responses. Indeed, disorders of the Fas/FasL apoptosis system in peritoneal immune cells, leading to reduced macrophage apoptosis, have been suggested as a mechanism underlying excessive macrophage accumulation in endometriosis. Our previous work [47] pointed to apoptotic impairments resulting in macrophage overactivation, possibly contributing to ectopic endometrial cell survival. These processes have been discussed in many studies (e.g., by Olive et al. [48], Halme et al. [49], Hammond et al. [50], and Koutsilieris et al. [51]). It remains unclear whether immune dysfunction is a cause or consequence of endometriosis; however, a large body of literature suggests that the observed immune changes actively contribute to pathogenesis.

Some immunological factors highlighted in prior studies appear to be involved in the pathogenesis of endometriosis and were also observed in our study. For instance, we detected a significant upregulation of IL‐1α in the PF of the endometriosis group. Elevated IL‐1 (particularly IL‐1β) levels in endometriosis have been reported by others and are thought to contribute to inflammation, angiogenesis, and pain generation [52]. In our study, IL‐1α levels were higher in endometriosis, even when considering the minimum and maximum values, compared to the reference values, reinforcing the notion of a proinflammatory peritoneal milieu. This finding reinforces the notion of a proinflammatory peritoneal milieu in patients with endometriosis. Interestingly, this elevation in IL‐1α was especially pronounced in many early‐stage cases (Stages I–II), which is in line with some reports suggesting that cytokine disturbances can be evident even in minimal disease [52]. Sikora et al. [53] also reported elevated IL‐1 family cytokine activity in endometriosis, describing an impairment of the IL‐1 network. They noted a delicate balance between “good” and “bad” IL‐1 effects, suggesting that differing concentrations of IL‐1α, pro–IL‐1β, and mature IL‐1β might shift inflammation from acute to a chronic profile. Our data support the idea that high IL‐1α levels in PF could favor chronic inflammation in endometriosis. Notably, macrophages in endometriosis may release more IL‐1α (which can act in an autocrine and paracrine fashion) despite counter‐regulatory mechanisms such as soluble IL‐1 receptors or IL‐1 receptor antagonists (IL‐1ra).

Second, we found approximately a twofold lower concentration of IL‐12 in the PF of patients with endometriosis than in that of the controls. These results suggest that a lack of IL‐12 may contribute to the reduced cytotoxic activity of T lymphocytes and NK cells in endometriosis. IL‐12 is a pivotal cytokine for Th1 immune responses; insufficient IL‐12 could lead to lower IFN‐γ secretion, impaired NK cell activation, and diminished elimination of ectopic cells [23, 24]. Previous studies have documented decreased NK cell activity in the PF of women with endometriosis [54, 55]. Ho et al. [56] found significantly lower IFN‐γ production in endometriosis PF, along with impaired T‐cell proliferation, aligns with the concept of inadequate IL‐12‐driven activation. In our study, the correlation between IL‐12 levels and macrophage percentage was negative but did not reach statistical significance (*ρ* = −0.246, *p* > 0.05), and an equivalent nonsignificant, near‐zero correlation was found in the reference group. These data therefore do not demonstrate that macrophage abundance or activity drives the reduction in IL‐12, and we present this link as a biological hypothesis rather than a conclusion supported by our correlation analysis. One plausible, hypothesis‐generating mechanism is that local immune regulation in the PF could suppress IL‐12 production as macrophage numbers or activity increase. If diminished IL‐12 in endometriosis reflects a genuine immunological defect, it could in principle allow ectopic endometrial lesions to evade cytotoxic immune surveillance, since IL‐12 facilitates clearance of aberrant cells by stimulating cytotoxic lymphocytes. Testing this hypothesis directly will require larger cohorts with sufficient power to detect modest correlations and, ideally, functional assays of macrophage–cytokine crosstalk rather than cross‐sectional concentration data alone.

It is important to consider how our results compare with those of previous studies on PF cytokines in endometriosis, as their findings have not always been consistent. For IL‐12, earlier investigations have reported mixed results. Zeyneloglu et al. measured IL‐12 levels in PF and found no significant difference between women with and without endometriosis; IL‐12 concentrations were low in both groups (∼1.1 pg/mL in endometriosis vs. 1.6 pg/mL in controls) [57]. They concluded that IL‐12 was detectable but not altered by the disease. In contrast, Fairbanks et al. (2009) observed that IL‐12 levels were higher in endometriosis, particularly in patients with advanced‐stage disease, than in controls [58]. Their study of 72 patients with endometriosis and 33 controls showed a significant increase in IL‐12 levels in the PF with increasing disease severity. Our current finding of decreased IL‐12 levels in endometriosis aligns more closely with the Zeyneloglu et al. [57] report and suggests that the patient population or disease stage may influence these results. Most of our endometriosis cases were early stage (Stages I–II), which could explain why our IL‐12 levels were not elevated as Fairbanks et al. [58] observed in severe cases. Interpretation of stage‐specific cytokine patterns should be approached cautiously because of the relatively small number of patients within each rASRM subgroup, particularly Stage IV disease. Although early‐stage disease (Stages I–II) represented more than half of the endometriosis cohort, the number of advanced‐stage cases was insufficient for adequately powered subgroup analyses. Therefore, no formal comparisons between early and advanced disease stages were performed. It is also possible that methodological differences (e.g., assay sensitivity or timing in the menstrual cycle) contributed to the discrepancies among studies. These may include variations in disease stage distribution, timing of PF collection during the menstrual cycle, patient ethnicity, sample handling protocols, assay sensitivity, and the definition of control groups. Importantly, many earlier studies used laparoscopically negative controls without histological exclusion of microscopic disease, which may have contributed to inconsistent cytokine profiles. Differences in ELISA kits, detection thresholds, and exclusion criteria for blood‐contaminated samples may also partially explain discrepancies between studies. For example, Gazvani et al. [59] found no difference in PF IL‐12 levels after accounting for menstrual cycle phase and disease stage. In our study, all samples were collected during the follicular phase, and samples with visible blood contamination were predefined for exclusion, which minimized potential variability and confounding effects. In addition, highly sensitive ELISA assays with low pg/mL detection limits were used, thereby reducing the likelihood that biologically relevant differences were missed because of technical insensitivity. These methodological considerations strengthen the reliability of the observed decrease in IL‐12 concentrations despite the relatively small sample size of this pilot study.

For IL‐1, many studies have focused on IL‐1β or related cytokines (such as IL‐18 or IL‐1ra) rather than IL‐1α. Sikora et al. (2018) noted an imbalance in the IL‐1 family in endometriosis, including elevated IL‐1β activity and reduced IL‐1ra, indicating a tilt toward a proinflammatory state [53]. Our study is one of the few to specifically examine IL‐1α levels in PF. We found that IL‐1α was elevated in endometriosis, which complements Sikora et al.’s findings on IL‐1β and suggests that both IL‐1α and IL‐1β (proinflammatory IL‐1 isoforms) are upregulated in endometriotic PF. Evidence from functional studies supports the importance of IL‐1 in this disease. For instance, in endometriotic stromal cell cultures, IL‐1 can promote the production of factors that contribute to lesion growth and innervation [60]. Moreover, Wu et al. provided indirect evidence of macrophage priming in endometriosis; they showed that peritoneal macrophages from women with endometriosis (especially in advanced stages) produced higher levels of IL‐6, IL‐10, and IL‐12 upon LPS stimulation than those from controls [61]. Although Wu et al. did not measure IL‐1 in their study, their findings of hyper‐responsive macrophages corroborate our observation of an immune cell activation state that includes robust IL‐1α production in vivo. The heightened cytokine secretion capacity of endometriosis‐associated macrophages likely contributes to the elevated IL‐1α levels we detected.

In addition, a recent study by Smycz‐Kubańska et al. expanded the scope of IL‐1 family cytokines implicated in endometriosis. They measured multiple IL‐1 family members (IL‐36α, IL‐36β, IL‐36*γ*, IL‐36Ra, IL‐37, and IL‐38) in PF and found that all tested IL‐1 family cytokines were significantly increased in patients with endometriosis compared to controls [35], with levels correlating with disease severity. This underscores the broad activation of the IL‐1 cytokine family in the peritoneal environment of endometriosis. Our current study, which focuses on classic IL‐1α, complements these findings by demonstrating that even the traditional IL‐1 axis is skewed toward a proinflammatory state in endometriosis. Unlike Smycz‐Kubańska et al., who studied newer members of the IL‐1 family, we provide novel data on IL‐1α, reinforcing that the entire IL‐1 family network (from IL‐1α/β to IL‐36 and IL‐37) is dysregulated in endometriosis. Together, these comparisons illustrate that while some cytokine patterns (e.g., high IL‐1 and related mediators) are consistently observed, others, such as IL‐12, have been variable across studies, possibly due to differences in patient selection, endometriosis stage, or technical factors, as discussed above. Notably, in a multiplex analysis of 48 peritoneal fluid cytokines in women evaluated for infertility, Jørgensen et al. identified a distinct endometriosis‐associated profile (elevated IL‐8, MCP‐1, HGF and SCGF‐β, and reduced IL‐13), consistent with a dysregulated Th1/Th2 response [62].

Overall, our findings of high IL‐1α and low IL‐12 levels in endometriosis PF support the concept of immune imbalance in this disease. Elevated IL‐1α levels indicate an exaggerated inflammatory response, which could facilitate processes such as angiogenesis and nerve fiber sensitization, contributing to lesion growth and pain [37]. Deficient IL‐12 suggests impaired cell‐mediated immunity, potentially explaining the well‐documented reduction in NK cell cytotoxicity in endometriosis [56]. This combination—a proinflammatory, proangiogenic push (via IL‐1α and others) and weakened antiectopic immune surveillance (via low IL‐12 and downstream IFN‐γ)—creates a peritoneal environment conducive to the establishment and persistence of endometriotic implants. Current evidence strongly indicates that immune cells (such as macrophages) and the cytokines they produce actively contribute to the acceleration of implantation and growth of endometrial tissue in ectopic locations [59]. Our observation of a significantly higher percentage of macrophages in endometriosis PF supports the hypothesis that these immune components promote disease development. It is worth noting that in our study, we did not distinguish peritoneal macrophage subtypes (M1 inflammatory vs. M2 regulatory); an imbalance between these subsets could be important in local inflammation and tissue remodeling. Prior research suggests that endometriotic macrophages often exhibit an altered phenotype that is not purely M1 or M2; however, further work is needed in this area [33, 34]. Future studies should explore macrophage polarization in endometriosis and its relationship with IL‐1 and IL‐12 production.

### 4.1. Limitations

This study has several limitations. First, the sample size was relatively small (*n* = 47), reflecting the pilot nature of the investigation and limiting the statistical power of subgroup analyses. In particular, the relatively small number of advanced‐stage cases prevented adequately powered stage‐specific comparisons. Second, the control group consisted of women with idiopathic infertility rather than healthy fertile controls. Although no visible endometriotic lesions were observed during laparoscopy, random peritoneal biopsies were not performed; therefore, occult microscopic disease cannot be completely excluded. Consequently, the reference group should be interpreted as laparoscopically negative rather than histologically confirmed disease‐free. This limitation may have introduced misclassification bias and should be considered when interpreting between‐group cytokine differences.

Third, although most endometriotic lesions were histologically confirmed, not all superficial Stage I lesions underwent biopsy because of their small size and typical laparoscopic appearance. Histopathological confirmation remains the diagnostic gold standard, and incomplete confirmation may represent an additional source of diagnostic uncertainty.

Fourth, all samples were collected during the follicular phase of the menstrual cycle. Although this approach minimized hormonal variability, the study could not assess potential cycle‐dependent fluctuations in cytokine concentrations.

Fifth, variability in PF volume may have influenced cytokine concentrations through dilution effects because cytokine levels were expressed per mL of fluid and were not normalized to total aspirated volume.

Sixth, several potentially relevant clinical variables, including body mass index (BMI), pain severity scores, duration of infertility, previous pelvic surgery, and detailed medication history, were not systematically available in the original study database and therefore could not be included in the analysis. These factors may influence the clinical presentation and inflammatory profile of endometriosis and should be considered in future studies. This is particularly relevant for BMI, given the broader problem of biomarker heterogeneity in endometriosis research: recent data on the leptin/BMI ratio in plasma and PF suggest that adipokine‐related markers may show compartment‐ and phenotype‐dependent associations with endometriosis, but their stand‐alone diagnostic utility remains limited and requires further validation [63]. Because adiposity can independently influence systemic and local inflammatory tone, the absence of BMI data in the present cohort represents a specific, unaddressed source of potential confounding for the IL‐1α and IL‐12 differences reported here, and future studies should explicitly account for BMI and adipokine status when interpreting cytokine‐based biomarkers of endometriosis.

Finally, only two cytokines (IL‐1α and IL‐12) were analyzed. Endometriosis is characterized by a complex inflammatory network involving multiple cytokines, chemokines, and immune cell populations. Therefore, broader immune profiling and functional immune assays were beyond the scope of this pilot study.

### 4.2. Future Directions

Given these limitations, future research should address them with more comprehensive and larger studies. A priority would be to analyze a larger cohort of patients, including those with all stages of endometriosis, and a more diverse control group (for instance, fertile women undergoing tubal ligation could serve as true healthy controls [26]). This would help validate whether the IL‐1α↑/IL‐12↓ profile holds true across a broader population and whether it correlates with clinical parameters (e.g., pain severity, infertility, and lesion burden). Additionally, investigating the functional polarization of peritoneal macrophages in endometriosis is an important next step. Characterizing the proportion of classically activated (M1) and alternatively activated (M2) macrophages and their respective cytokine production profiles in patients with endometriosis could clarify how the immune environment shifts in this disease. Such studies could employ advanced techniques, such as expanded flow cytometry panels or single‐cell RNA sequencing, to delineate macrophage subpopulations and their activation status in the PF [33, 34].

Another promising avenue is the exploration of therapeutic interventions targeting these immune factors. Our findings and those of others suggest that blocking IL‐1 activity or enhancing IL‐12 activity may modulate the disease. A pilot clinical trial has tested the use of an IL‐1 receptor antagonist (anakinra) in endometriosis, with some encouraging results [36]. Further clinical trials are needed to assess the efficacy of IL‐1 blockade in reducing endometriosis‐related inflammation and pain. However, strategies to boost the Th1/NK cell response (for example, by augmenting IL‐12 or IFN‐γ activity) could be envisioned. Direct IL‐12 therapy in humans requires caution due to potential side effects; however, local or targeted approaches may be possible. Notably, an animal study by Itoh et al. showed that the administration of IL‐12 in a mouse model suppressed the development of endometriotic lesions via NK cell activation [25]. This raises the question of whether enhancing IL‐12‐dependent pathways could help human patients, and future research should investigate indirect ways to achieve this (such as drugs that stimulate endogenous IL‐12 production or bolster NK cell function). Moreover, broader immune modulation strategies, such as promoting a shift from M2 to M1 macrophages or inhibiting macrophage recruitment (e.g., via MCP‐1 inhibitors), may be therapeutic avenues worth exploring. Such approaches would benefit from a deeper understanding of the immune milieu in endometriosis, for instance, simultaneous profiling of pro‐ and anti‐inflammatory cytokines, growth factors, and chemokines in the PF and serum of patients with the disease. Local (PF) and systemic (plasma) concentrations of endometriosis‐associated biomarkers do not always change in parallel: compartmentalized inflammation, differential clearance, and dilution effects can uncouple peritoneal from circulating cytokine levels, so that a marker’s discriminatory performance in PF cannot automatically be assumed to generalize to plasma, or vice versa. This point is illustrated by a recent study that measured the inflammation‐associated protein Annexin A2 simultaneously in plasma and PF of women with and without endometriosis and found differing discriminatory performance between the two compartments [64]. Future studies of IL‐1α and IL‐12 in endometriosis would therefore benefit from paired plasma and PF sampling within the same patients, which would clarify whether the peritoneal IL‐1α↑/IL‐12↓ pattern observed here is also reflected systemically or is instead a compartment‐specific phenomenon, with direct implications for the feasibility of a minimally invasive, blood‐based biomarker.

In conclusion, our pilot study provides evidence of an immunological imbalance in endometriosis, characterized by elevated IL‐1α and diminished IL‐12 levels in the peritoneal cavity. These findings reinforce the concept that endometriosis has a significant immune component, wherein an inflammatory phenotype prevails, and immune surveillance is dampened. The imbalance between a proinflammatory cytokine (IL‐1α) and a key immunoregulatory cytokine (IL‐12) suggests a peritoneal environment that could favor the implantation and persistence of endometriotic lesions. Future studies should build on these results by enrolling larger patient populations, utilizing more comprehensive immunological assays, and investigating targeted therapies. By doing so, we can move closer to identifying immune biomarkers for endometriosis and developing novel treatments that address the immune dysregulation at the root of this disease.

## 5. Conclusions

Women with endometriosis exhibited a significantly higher proportion of PF macrophages than the reference group of women with idiopathic infertility and no macroscopic signs of endometriosis, supporting the concept of immune cell accumulation within the peritoneal cavity. PF IL‐1α concentrations were significantly elevated, whereas IL‐12 concentrations were significantly reduced in patients with endometriosis compared with this reference group. As random peritoneal biopsies were not performed in the reference group, occult microscopic endometriosis cannot be fully excluded, and these women should accordingly be regarded as laparoscopically, rather than histologically, disease‐free. The imbalance between IL‐1α and IL‐12 suggests a peritoneal microenvironment characterized by enhanced inflammation and impaired cytotoxic immune activity, which may contribute to the establishment and persistence of endometriotic lesions. The exploratory ROC‐based cutoffs identified for macrophage percentage, IL‐1α, and IL‐12 reflect apparent, in‐sample discriminatory performance in this single pilot cohort and have not been externally validated. They should be regarded as hypothesis‐generating only, and no claim of current clinical usefulness is made for these thresholds.

No statistically significant correlations were observed between cytokine concentrations and macrophage percentage in either study group. Therefore, the observed alterations in IL‐1α and IL‐12 levels should be interpreted independently of direct cytokine–macrophage associations within this cohort.

Although derived from a pilot study, these findings further support the involvement of macrophage‐related immune dysregulation in the pathophysiology of endometriosis. Larger studies incorporating broader immunological profiling are required to validate these observations and to determine whether immune‐targeted therapeutic approaches, including modulation of IL‐1α‐ or IL‐12–related pathways, may have clinical relevance in the management of endometriosis.

## Author Contributions

Monika Kopeć: conceptualization, data curation, investigation, methodology, visualization, writing–original draft, and writing–review and editing. Małgorzata Wojtaś: data curation and writing–original draft. Aleksandra Kamińska: data curation and methodology. Michał Kos: data curation. Jan Kotarski: methodology and writing–original draft. Weronika Grochowska: visualization and writing–original draft. Katarzyna Dos Santos Szewczyk: writing–review and editing. Marek Gogacz: conceptualization, investigation, methodology, supervision, and writing–review and editing.

## Funding

This article has no funding.

## Ethics Statement

The study was conducted in accordance with the Declaration of Helsinki and approved by the Bioethics Committee of the Medical University of Lublin (KE‐0254/290/2003) for studies involving humans.

## Conflicts of Interest

The authors declare no conflicts of interest.

## Data Availability

The datasets used in this study are available from the corresponding authors upon reasonable request.
